# Momentum-dependent scaling exponents of nodal self-energies measured in strange metal cuprates and modelled using semi-holography

**DOI:** 10.1038/s41467-024-48594-6

**Published:** 2024-05-29

**Authors:** S. Smit, E. Mauri, L. Bawden, F. Heringa, F. Gerritsen, E. van Heumen, Y. K. Huang, T. Kondo, T. Takeuchi, N. E. Hussey, M. Allan, T. K. Kim, C. Cacho, A. Krikun, K. Schalm, H.T.C. Stoof, M. S. Golden

**Affiliations:** 1 Van der Waals - Zeeman Institute, Institute of Physics, University of Amsterdam, Sciencepark 904, 1098 XH Amsterdam, The Netherlands; 2 Institute for Theoretical Physics and Center for Extreme Matter and Emergent Phenomena, Utrecht University, Utrecht, The Netherlands; 3 Institute for Solid State Physics, University of Tokyo, Kashiwa, Chiba 277-8581 Japan; 4 Energy Materials Laboratory, Toyota Technological Institute 2-12-1 Hisakata Tempaku-ku, Nagoya, 468-8511 Japan; 5 High Field Magnet Laboratory (HFML-EMFL) and Institute for Molecules and Materials, Radboud University, Toernooiveld 7, 6525 ED Nijmegen, The Netherlands; 6 H. H. Wills Physics Laboratory, University of Bristol, Tyndall Avenue, Bristol, BS8 1TL UK; 7 Leiden Institute of Physics, Leiden University, Leiden, The Netherlands; 8 Diamond Light Source, Harwell Campus, Didcot, OX11 0DE UK; 9 NORDITA, KTH Royal Institute of Technology and Stockholm University, Hannes Alfvéns väg 12, 106 91 Stockholm, Sweden; 10 Institute-Lorentz for Theoretical Physics, Leiden University, P.O. Box 9506 Leiden, The Netherlands; 11 Dutch Institute for Emergent Phenomena (DIEP), Sciencepark 904, 1098 XH Amsterdam, The Netherlands

**Keywords:** Electronic properties and materials, Superconducting properties and materials, Theoretical physics

## Abstract

The anomalous strange metal phase found in high-*T*_*c*_ cuprates does not follow the conventional condensed-matter principles enshrined in the Fermi liquid and presents a great challenge for theory. Highly precise experimental determination of the electronic self-energy can provide a test bed for theoretical models of strange metals, and angle-resolved photoemission can provide this as a function of frequency, momentum, temperature and doping. Here we show that constant energy cuts through the nodal spectral function in (Pb,Bi)_2_Sr_2−*x*_La_*x*_CuO_6+*δ*_ have a non-Lorentzian lineshape, consistent with a self-energy that is k dependent. This provides a new test for aspiring theories. Here we show that the experimental data are captured remarkably well by a power law with a *k*-dependent scaling exponent smoothly evolving with doping, a description that emerges naturally from anti-de Sitter/conformal-field-theory based semi-holography. This puts a spotlight on holographic methods for the quantitative modelling of strongly interacting quantum materials like the cuprate strange metals.

## Abstract (web summary)

Strange metal behaviour of high-Tc superconductors, characterised by unconventional electrical and thermodynamic properties, still poses challenges for theory. Smit et al. report experimental features in the self-energy of a strange metal that are consistent with predictions by holographic theoretical methods.

## Introduction

The rich temperature versus doping diagram of the cuprate high-*T*_*c*_ superconductors presents a cornucopia of non-conformity^1^. The superconductivity does not follow BCS theory anywhere in the phase diagram^2,3^, and in their normal state these unusual metals display qualitatively different experimental behaviour compared to the Fermi liquid, with the linear-in-*T* resistivity and quadratic-in-*T* Hall angle being among the most strange.

One intriguing possible explanation of strange metal phenomenology is that it is controlled by a strongly interacting quantum critical *phase*. If so, the question follows whether their properties can be described by holographic emergence principles, as these approaches are uniquely suitable for describing such physics. Evidence for a quantum critical phase comes from high magnetic-field electrical transport experiments on overdoped cuprates^4,5^, and from laser-ARPES on Bi_2_Sr_2_CaCu_2_O_8+*δ*_ (Bi-2212)^6^.

Here, we exploit the low *T*_*c*_ and simple, single-band nature of (Pb,Bi)_2_Sr_2−*x*_La_*x*_CuO_6+*δ*_ ([Pb,Bi]-2201) to access the normal state over a wide range in *ω* and *T* using high-resolution ARPES. We show that the constant energy cuts through the nodal spectral function (MDCs) have an increasingly non-Lorentzian lineshape as *k* moves away from *k*_*F*_. Unable to successfully model the observed MDC asymmetry as arising from potential trivial causes (see Supplementary Notes 2–6), including doping inhomogeneity and local surface structural facetting, we argue the observations are consistent with a self-energy of the electrons being not only a function of *ω* and *T*, but also dependent on the magnitude of the momentum away from the Fermi momentum *k* − *k*_*F*_.

After presenting the experimental data, we switch gears to test whether a quantitative theoretical description can be found using the tools of the holographic duality known as the Anti-de Sitter/Conformal Field Theory correspondence (AdS/CFT) from string theory^7,8^. AdS/CFT approaches have been shown to successfully capture both Fermi-liquid-like^9^ and non-Fermi-Liquid-like aspects of strange metallic behaviour^10^, including power-law self-energies with smoothly varying scaling exponents^11,12^.

Adopting a semi-holographic theoretical treatment^13^, whose details are given in the Methods section, we can match its predicted *k*-dependent spectral function to the high-precision ARPES data across the doping range studied, for a wide energy range below the Fermi level *E*_*F*_ and for momenta well away from *k* − *k*_*F*_.

Here we show that, firstly, our ARPES data confirm that nodal electronic self-energies display a power law in frequency and temperature. Secondly, momentum-dependent scaling exponents describing the self-energies give an excellent match to the experimental data. Thirdly, the observed exponents at *k*_*F*_ grow smoothly from unity at optimal doping towards the Fermi-liquid value of two, though this is never reached, even for non-superconducting samples. These results further strengthen the notion that the optimally and overdoped Bi-based strange metals do represent a novel quantum critical phase.

## Results

### Nodal ARPES data: power-law analysis with *k*-independent self-energy

**Fig. 1:**
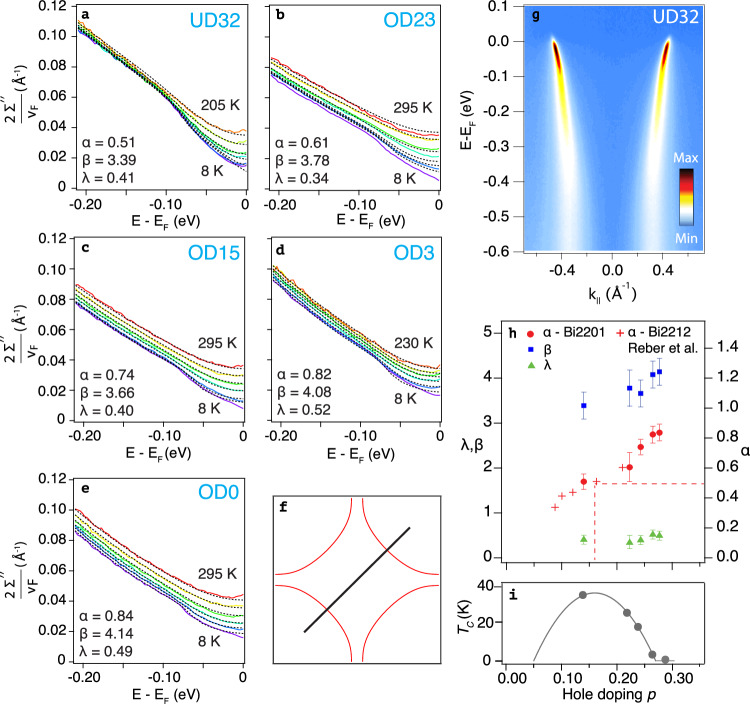
Nodal self-energy of single-CuO_2_-layer (Pb,Bi)-2201. **a**–**e** Temperature-dependent self-energies from ARPES for five different doping levels, extracted using symmetric Voigt fits to MDCs, plotted using colour-coded solid lines for temperature. The dashed, black lines are results to two-dimensional (in *T* and *ω*) fits employing three parameters (*α*, *β*, *λ*), using the power-law-liquid formalism introduced in Ref. ^6^ and given in Eq. (1). Fitting parameters used are indicated in each data panel, and gathered together with the analogous parameters for Bi-2212^6^ in (**h**). The red dashed lines indicate the marginal Fermi liquid (*α*  = 0.5 at optimal doping), which is shown above the canonical Presland dome (**i**) used to determine the doping level of the measured samples^18^. **g** Shows a typical measured ARPES dataset, containing both the positive-*k* and negative-*k* nodal branches, along the *k*-space direction indicated in the schematic Fermi surface in (**f**). Supplementary Table 1 in the Supplementary Note 7 lists all temperatures measured for each doping level. The error bars in (**h**) reflect the sensitivity of *α*, *β* and *λ* to details of the fitting procedure, in particular the energy and temperature range over which the fits are performed.

In Fig. 1 we show the imaginary part of the nodal self-energy of (Pb,Bi)_2_Sr_2−*x*_La_*x*_CuO_6+*δ*_ over a large range in frequency, doping and temperature. The commonly adopted assumption is made that the bare dispersion is linear, *ε*(*k*) = *v*_*F*_(*k* − *k*_*F*_), with *v*_*F*_ being the Fermi velocity and *k*_*F*_ the Fermi wave vector. Under the crucial, additional assumption of negligible *k* dependence for the self-energy - Σ(*k*, *ω*) ≃ Σ(*ω*) - the single-particle spectral function reduces to a symmetric, Lorentzian lineshape as a function of momentum *k* at each fixed frequency *ω*, i.e., $L(k)=\frac{W}{\pi }\frac{\Gamma /2}{{(k-{k}_{*})}^{2}+{(\Gamma /2)}^{2}}$. Here *W*(*ω*) is the intensity, k_*_(*ω*) the peak position and Γ(*ω*) its width. For the results presented in the rest of this paper, *W*(*ω*) is not a key parameter and will not be discussed further. In practice, the peaks are better fit using a Voigt lineshape, the Gaussian part of which accounts for experimental resolution. In this framework, the imaginary part of the self-energy is then proportional to the width of the Lorentzian component as Σ^*″*^(*ω*) = *v*_*F*_Γ(*ω*)/2^14^.

The extraction of the self-energy from these ARPES MDC widths can then be approached in different ways. One is to fit the data using pre-defined scenarios for the (*ω*, *T*)-dependence of the self-energy, such as linear- or quadratic-in-(*ω*, *T*) behaviour^14–16^. Another is to allow the power-law exponent to take on whichever value best matches the data at that doping level, conducting fits working simultaneously in frequency and temperature space^6^.

In Fig. 1, we show the results of a first benchmarking of our (Pb,Bi)_2_Sr_2−*x*_La_*x*_CuO_6+*δ*_ ARPES data, adopting the latter approach, in which the self-energy is given by Eq. (1), which contains three dimensionless parameters *α*, *β* and *λ*:

1 $$\Gamma=\frac{2{\Sigma }^{{''} }(\omega,T)}{{v}_{F}}={G}_{0}(\omega,T)+\lambda \frac{{[{(\hslash \omega )}^{2}+{(\beta {k}_{B}T)}^{2}]}^{\alpha }}{{(\hslash {\omega }_{N})}^{2\alpha -1}}.$$

Here *λ* is a coupling constant describing the strength of the interaction, normalised to an energy scale *ℏω*_*N*_ = 0.5eV for all dopings and the parameter *β* sets the balance between the relative influence of temperature and frequency. *G*_0_(*ω*, *T*) is an extra term, combining a self-energy contribution from electron-phonon coupling, that is most clearly seen at low T around an energy of 70 meV, with impurity scattering, described in Supplemenary Note 1.

Note that Eq. (1) - which has been dubbed the power-law liquid, or PLL - describes the marginal Fermi liquid^17^ with a power of unity (*α* = 1/2) at optimal doping and the quadratic temperature and frequency behaviour of a Fermi liquid emerges when *α* = 1.

Figure 1a–e show the experimentally extracted self-energies (coloured lines), together with the result of a two-dimensional (*ω*, *T*) fit to the data using Eq. (1) (dashed lines). An overview of the parameters extracted from the fits is given in Fig. 1h, together with the parameters previously determined for Bi-2212^6^. Compared to the latter, the ARPES data presented here cover a complementary doping range from near optimal doping (*p* = 0.14) to such overdoping that superconductivity disappears and also includes doping levels on either side of p* ^18^, showing that neither superconductivity nor the opening of the pseudogap influence the observed trend in the nodal self-energy. Across this doping interval, this analysis shows the exponent to increase smoothly from 1.02 (*α* = 0.51) - consistent with the marginal Fermi liquid expectation - to 1.68 (*α* = 0.84) for the highest hole doping measured. This means that even at the edge of the superconducting dome, the quadratic power of the Fermi liquid (*α* = 1) is not reached.

The remarkable degree of continuity in the *α* values evident in Fig. 1h highlights good agreement between the nodal self-energy behaviour in both single- and bi-layer cuprates.

### Asymmetric ARPES MDCs: evidence of *k*-dependent power-law exponents

**Fig. 2:**
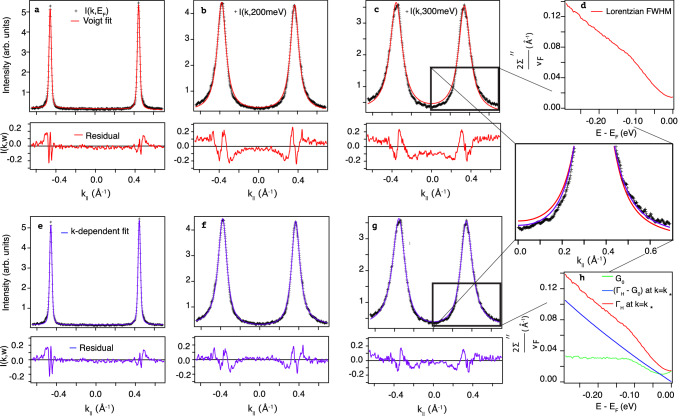
Closer look at MDC lineshapes for UD32 nodal ARPES data. **a**–**c** A trio of MDCs at energies indicated measured at 8K, with symmetric peak fits in red. The residuals grow as the binding energy grows. Here (**d**) shows the resulting 2Σ^*″*^(*ω*)/*v*_*F*_ = Γ(*ω*) from the symmetric fits. **e**–**g** The same three MDCs now fit in purple using our model given by Eq. (3), including the holographically-predicted *k* dependence, with *V* set to −1. The residuals are clearly superior to the symmetric fit for energies further from *E*_*F*_. **h** The imaginary part of the self-energy 2Σ^*″*^(*k*, *ω*)/*v*_*F*_ = Γ_*H*_(*k*, *ω*) at k_*_(*ω*) (red), which includes the *k*-dependent self-energy (blue) and the free fitting parameter *G*_0_ (green).

Crucial to the analysis just carried out is that the MDC lineshape of each nodal branch is a symmetric Lorentzian over an extended range of frequency, signalling negligible *k* dependence of the self-energy. In Fig. 2a–d we show an examination of the Lorentzian fits in detail.

Figure 2a ﻿shows that a Lorentzian spectral function gives a very good fit close to *E*_*F*_, and yields a small residual. However, at higher binding energy, such as at 200 and 300 meV, the foot of the peaks clearly show that the data (black crosses) are not adequately captured using two symmetric peaks (red lines). The experimental bottom line is that the MDC peaks in Fig. 2﻿b, c are asymmetric: showing more spectral weight at large momenta ∣*k*∣ > ∣k_*_(*ω*)∣, compared to ∣*k*∣ < ∣k_*_(*ω*)∣, thus leading to residual values of differing signs inside and outside the MDC peak pair.

In the Supplementary Information, we discuss in turn a number of possible ‘trivial’ sources that could lead to non-Lorentzian MDCs. These include non-zero curvature of an underlying ‘bare band’ (Supplementary Note 2), averaging over local doping variations or local facetting at the sample surface (Supplementary Note 3), inhomogeneous detector response (Supplementary Note 4), or contributions from background signals (Supplementary Notes 5 and 6). None of these are able to adequately reproduce the observed asymmetry of the MDC lineshapes (reduced/increased intensity on the low/high ∣*k*∣ side of the peak) and that this asymmetry grows with *k* − *k*_*F*_. Therefore, after this due diligence, we propose a simple yet profound explanation of the MDC asymmetry as being an expression of *k* dependence of the electronic self-energy itself.

One model-independent approach to describe this would be to linearly expand the measured self-energy around *k*_*F*_, reported in Supplementary Fig. 10. This significantly enlarges the set of fitting parameters, by means of an additional frequency and temperature dependent function. In the following we show that theoretical input provided by AdS/CFT enables the data to be fitted across the full frequency range as proficiently as those near *E*_*F*_ could be, all without any expansion in the number of free parameters.

### Semi-holographic theoretical description

Physically, a semi-holographic model describes an electron that interacts with a CFT (accounting for a quantum critical state deformed by non-zero *T* and chemical potential) via linear coupling to a fermionic operator ${{{{{{{\mathcal{O}}}}}}}}$ that has a unique scaling dimension. As a result, the self-energy becomes proportional to the correlation function $\langle {{{{{{{{\mathcal{OO}}}}}}}}}^{{{{\dagger}}} }\rangle (k,\omega,T)$ in the CFT. The latter can be determined from the holographic dictionary^7,8^, and automatically inherits certain scaling properties from the ‘critical’ CFT. To be applicable to the cuprate strange metals, the model must have a dynamical critical exponent *z* → *∞* in order to recover the behaviour that is well-described by Eq. (1) when *k* is close to *k*_*F*_. From this imposition of local quantum criticality, there follows a fundamental condition from the theory side that the scaling exponents have to be momentum dependent^19^.

Here, for the CFT we use an Einstein-Maxwell-Dilaton model of holography, specifically the Gubser-Rocha model^20,21^, as it offers an analytical treatment of the gravitational spacetime. In the long-wavelength limit, within the framework of an emergent particle-hole symmetry, our semi-holography model then gives at *T* = 0:

2a $$\frac{2{\Sigma }^{{''} }(k,\omega )}{{v}_{F}}=\lambda \frac{{[{(\hslash \omega )}^{2}]}^{\alpha (k)}}{{(\hslash {\omega }_{N})}^{2\alpha (k)-1}}\,,$$

2b $$\alpha (k)=\alpha \left(1-\frac{k-{k}_{F}}{{k}_{F}}\right).$$

The semi-holographic self-energy can also be generalised to non-zero temperature^19,21^, and under our conditions is well approximated by replacing (*ℏω*)^2^ with ${(\hslash \omega )}^{2}+{(\beta {k}_{B}T)}^{2}$ in Eq. (2a). This also eases comparison to the PLL in Eq. (1), and highlights the key new insight that as frequency increases, and *k* ≃ *k*_*F*_ − *ω*/*v*_*F*_ departs from *k*_*F*_, a *k* dependence emerges in the exponent describing the (*ω*, *T*)-dependence of the self-energy. For low frequencies, where *k* ≃ *k*_*F*_, one returns to Eq. (1) of the PLL. The behaviour predicted by holography should leave a clear experimental fingerprint, namely that the ARPES MDCs are asymmetric: the data shown in Fig. 2 show this is the case.

**Fig. 3:**
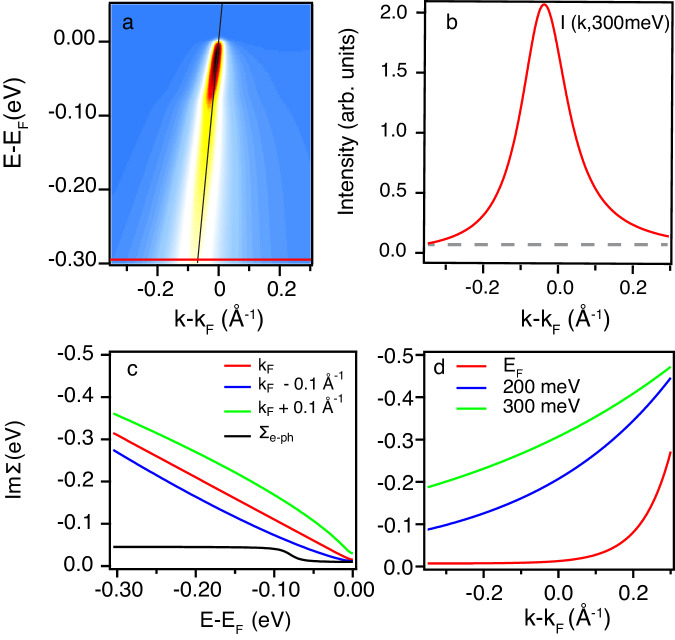
Simulated spectral function at optimal doping, using the *k*-dependent self-energy from semi-holography. **a** Spectral function with a linear bare band (v_*F*_ = 4 eV Å, k_*F* _= 0.45 Å^−1^), convoluted with the experimental *k* resolution and (**b**) MDC at non-zero binding energy generated using the full *ω* and *k*-dependent self-energy plotted in (**c**, **d**) from the semi-holographic model at *T*=20 K. Note that (**c**) includes the imaginary part of a *k*-independent phonon self-energy.

Figure 3a shows a simulated spectral function, generated using the semi-holographic self-energy shown in Fig. 3c, d. The resulting MDC asymmetry at non-zero energy is illustrated in Fig. 3b, visible as an increased intensity at the ∣*k*∣ > ∣k_*_∣ side of the peak maximum, just like in the experimental data of Fig. 2c. The next step is to test whether this holographic approach can yield superior quantitative fits to the ARPES data.

### Fits to the ARPES data inspired by the semi-holographic model

As non-Lorentzian MDCs force a major departure from conventional ARPES data analysis methodology, we now describe how to deal with this in the analysis of real data from (Pb,Bi)_2_Sr_2−*x*_La_*x*_CuO_6+*δ*_. The non-zero *T* version of the self-energy with the *k* dependence in Eq. (2b), suggests a modified fit-function *L*_*H*_(*k*) at the fixed *ω* and *T* relevant for each MDC:

3a $${L}_{H}(k)=\frac{W}{\pi }\frac{\frac{{\Gamma }_{H}}{2}}{{(k-{k}_{*})}^{2}+{\left(\frac{{\Gamma }_{H}}{2}\right)}^{2}},$$

3b $${\Gamma }_{H}(k)={G}_{0}+\lambda \frac{{[{(\hslash \omega )}^{2}+{(\beta {k}_{B}T)}^{2}]}^{\alpha (k)}}{\hslash {\omega }_{N}^{2\alpha (k)-1}},$$

3c $$\alpha (k)=\alpha \left(1+V\left[\frac{k-{k}_{F}}{{k}_{F}}\right]\right).$$

Γ_*H*_(*k*) in Eq. (3b) captures both the peak width and its asymmetry via momentum dependence built into the exponent *α*(*k*) in Eq. (3c). Moreover, the Gubser-Rocha holographic model discussed above in Eq. (2), actually requires that *V* = −1 for all frequencies. Therefore by fixing *α*, *β*, *λ* and *ω*_*N*_ to the PLL values at low energies and *V* to the value in the Gubser-Rocha model, only G_0_ in Eq. (3b) remains free to vary in the fitting process for each MDC. Then the resulting fit function in Fig. 2e–g has exactly the same number of free parameters as the PLL^6^.

We reiterate that the k-dependence of the exponent *α* describing the (*ω*, *T*)-dependence of the self-energy in Eqs. (3b, 3c) used to fit to the ARPES data follows directly from the semi-holographic prediction given in Eqs. (2a, 2b), itself a consequence of the requirement of local quantum criticality^19^.

Comparing Fig. 2b, c (PLL, *k*-independent) with Fig. 2f, g (*k*-dependent scaling exponents), it is clear that on adopting Eq. (3), the residuals in the panels f and g for energies well below *E*_*F*_ drop, becoming as low as they were for the PLL at *E*_*F*_. In particular, the zoom to Fig. 2c, g illustrates clearly that the asymmetry of the MDC peaks is now captured almost perfectly. We emphasise that the non-zero value of V, which accounts for the experimentally observed MDC asymmetry, is a non-trivial result and is deeply rooted in the holography. Figure 2h shows the total self-energy along the loci of the MDC peak-maxima in red, with the holography-inspired, k-dependent part in blue and the free fitting parameter G_0_(*ω*, *T*) in green. The latter can be seen to automatically take on the combined form of an offset (impurity scattering) plus a step function centred at the phonon energy of 70 meV.

### Testing the semi-holographic prediction

Having determined the free parameters in the Gubser-Rocha model (see Methods), the validity of the fits using Eq. (3) can be tested. For the Gubser-Rocha model adopted here, the asymmetry parameter should have a frequency-independent value of *V* = −1. The experimental data can now be used to test this in a second set of fits, now leaving *V* as a free MDC-fitting parameter for each *ω*. Figure 3 shows that—with no guiding restraint at all applied to *V*(*ω*)—the experimental data yield for all dopings, temperatures (see Supplementary Fig. 9) and frequencies a value of *V* in the vicinity of −1, which is close to the semi-holographic prediction.

The Gubser-Rocha model also yields values for *β*. In Fig. 1h, the experimental *β* value shows a lightly upward trend vs. hole doping in an interval between *β* = 3–4 for the doping levels studied. The Gubser-Rocha model itself (see Supplementary Fig. 9d) predicts a similar trend vs. doping, but with lower *β* values running between 2 and 3. A detailed derivation of parameters such as *β* falls outside the experimental focus of this paper, and will be presented in a separate publication.

**Fig. 4:**
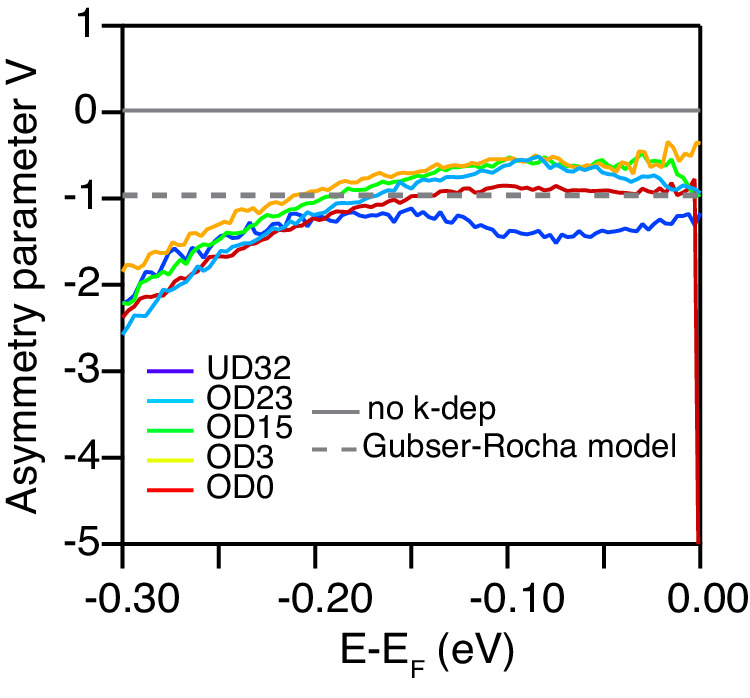
Testing the predictions from semi-holography. Asymmetry parameter *V* of the momentum-dependent scaling exponent extracted from the experimental ARPES data over the full doping range measured at 8K, obtained by performing MDC fits to the data using Eq. (3), where the leading scaling exponent *α*, the coupling constant *λ* and the quantity ${(\hslash \omega )}^{2}+{(\beta {k}_{B}T)}^{2}$ are fixed. A value *V* = 0 means there is no asymmetry, and *V* = −1 is the prediction from the semi-holographic Gubser-Rocha model. Data from analogous fits at high temperature are shown in Supplementary Fig. 9.

There is still some room for improvement, in particular as regards *β* output by the Gubser Rocha semi-holographic model. The spectral function fits to the ARPES data that are so successful in Fig. 2e–g used *β* values from the PLL parameterisation (not those directly predicted by the Gubser Rocha model), and the Gubser-Rocha inspired *V* = −1 in Eq. (3) admirably captures the asymmetry of the MDCs and when tested in Fig. 4 is found to be a stably representative value. These ARPES data and their parametrization presented here are an invitation to the many existing *z* = *∞* holographic models besides the analytical Gubser-Rocha model—or to completely different theoretical methods—to take the next step in capturing the modest frequency dependence of *V* seen in experiment, and the observed *β* values. We stress however that according to our experimental data, the non-zero value of *V* which underpins the observed MDC asymmetry must be accounted for by any theoretical approaches aiming to describe the spectral function of the nodal charge carriers in cuprates.

Before providing a discussion of other contexts in which *k*-dependent self-energies have been discussed, we re-iterate our main results. In the ARPES data of nodal carriers in the normal state of the single-layer cuprate (Pb,Bi)-2201, asymmetric, non-Lorentzian MDCs are observed for energies away from *E*_*F*_. As Supplementary Notes 2–6 show, we cannot explain this due to bare band non-linearity, doping inhomogeneity, surface roughness or technical problems with detector or backgrounds. Instead, analysing these in terms of the *ω* and *T* dependence of the electronic self-energy, an excellent fit for each doping level is obtained using a single power law with a *k*-dependent scaling exponent. Given our experimental data, power-law scaling is well supported over a significantly wider energy and temperature range than in ref. ^6^, and for the whole overdoped portion of the phase diagram. At *k* = *k*_*F*_, the *k*-dependent power-law exponent is found to take a nearly marginal Fermi-liquid value of unity (*α* = 0.51) at optimal doping, growing to 1.68 (*α* = 0.84) for the most overdoped, non-superconducting system studied. For *k* = *k*_*F*_, these results connect smoothly to and greatly extend those of Bi-2212 in Ref. ^6^. Significantly, for values *k* ≠ *k* − *k*_*F*_ these powers change. Such a *k* dependence is a key prediction from (semi)holographic models of strange metals as a quantum critical phase, and the excellent qualitative agreement between the experimental data and these family of models provides a benchmark for future work. In particular, any competing theoretical model for strange metals must be able to account for the observed ARPES MDC peak-asymmetry that is well described here by a momentum-dependent power-law self-energy.

In the cuprate context, numerous ARPES studies focus on the *k* dependence of the self-energy in terms of the *position* on the FS, both in the normal^15,22–24^ and superconducting states^25–27^. A dependence of the self-energy on *k* − *k*_*F*_ has been linked to the doping dependence of the high-energy velocity of the nodal states in ARPES^28^, that has been argued to possibly arise from strong electron-phonon coupling^29^. Moreover, asymmetric MDCs in ARPES data from LSCO have been proposed to be due to a *k*-dependent self-energy in phenomenological models based on ‘extremely correlated Fermi liquids’^30^ or have been proposed to be a more general signal of strong correlations^31^.

In a different condensed-matter realisation, *k*-dependent power laws have been observed in the zero-bias conduction anomaly in transport spectroscopy of nanowires^32^ and linked to non-linear Luttinger liquid theory^33^. One might therefore wonder if there is a link between the 1D physics in the nanowire case, and the carriers in the nodal *k*-space direction of the quasi-2D (Pb,Bi)-2201 system that seem well described by the 1D CFT encoded semi-holographically in the AdS_2_ infra-red geometry of the *z* = *∞* models.

The results presented here herald future comparisons of the experimentally-tested, theoretical spectral function to other experimental probes such as optical and DC conductivities^34^. Alternatively, many-body condensed-matter theories can be guided by both the experimental self-energies presented here and the results from the AdS/CFT analogue, opening up possible pathways out of phenomenology and into a microscopic understanding of strange metals.

## Methods

### ARPES measurements

All ARPES data presented here were recorded at beamline I05 of Diamond Light Source, using (horizontally) linearly polarised light at a photon energy of 28 eV. Due to this chosen photon energy we can combine excellent energy resolution (12meV) with enough range in *k* space to measure both the negative-*k* and positive-*k* nodal branches in a single *I*(*k*, *ω*) image (see Fig. 1a), which is very helpful in distinguishing the subtle effects at play from possible complicating factors such as signal background. Both a momentum and energy-dependent background have been taken into account during the analysis, as detailed in Supplementary Note 4. The energy resolution, together with the Fermi energy position - was confirmed by means of reference data from an amorphous Au film held in electrical contact with the sample. To account for resolution in the angular direction, both the Lorentzian MDC fit function used for the PLL analysis, and the L_*H*_ MDC fit function from Eq. (3) are convolved with a Gaussian of width 0.01 Å^−1^. In the symmetric PLL case this yields a Voigt lineshape as indicated in the main text and in Fig. 2.

All data were recorded in swept mode, to ensure any detector-response inhomogeneity is averaged out in the energy direction, and were also confirmed to be free of detector non-linearity effects^35^. All data were recorded in the XΓX direction in the Brillouin zone.

All the temperature-dependent data per sample have been acquired during a single heating cycle starting from base temperature. To prevent any effects of sample aging due to the changing temperature, extreme care was taken by very slow, controlled heating of the sample, so as to avoid large pressure jumps in the UHV environment. Select samples have been recooled and have had their nodal dispersion remeasured. The nodal 2k_*F*_ values and the minimum MDC width at the Fermi level are taken as confirmation of no sample degradation.

High quality single crystals of diffraction replica-free (Pb,Bi)_2_Sr_2−*x*_La_*x*_CuO_6+*δ*_, or (Pb,Bi)-2201, were grown using floating-zone techniques. Individual crystals were annealed in varying atmospheres for varying lengths of time, so as to change the oxygen content and with it the hole doping, controlling the carrier concentration and *T*_*c*_. The critical temperatures were determined either via resistivity or AC-susceptibility measurements and the doping level was read off using *T*_*c*_ and the Presland formula^36,37^. For the sample OD0K, *T*_*c*_ ≲ 2*K*$\lesssim 0.05{T}_{c}^{max}$.

### Holography and semi-holography

The Anti-de Sitter/Conformal Field Theory correspondence (AdS/CFT) links a classical gravitational theory with one additional spatial dimension to a strongly interacting quantum theory on the boundary of the extended space. In practice, it provides a systematic way to compute the response functions for a quantum theory with strong interactions, even at non-zero temperature and in the presence of a chemical potential, by means of solving a (relatively speaking) simpler problem, namely solving classical gravitational equations. For our purposes, therefore, the curved space is three dimensional and the quantum theory should describe the correlated physics of the maximally entangled fermions in the quasi-2D high-*T*_*c*_ cuprates^7,8^. To be precise, holography describes the physics of a composite fermion ${{{{{{{\mathcal{O}}}}}}}}$ with a large number of degrees of freedom, i.e., in the so-called large-*N* limit. However, as we here are interested in testing theoretical predictions against spectral functions obtained through ARPES data, we ultimately want to compute the response function for an elementary fermion *ψ*. The solution is found within the semi-holographic framework^13^, where the fermion *ψ* is linearly coupled to the composite fermion ${{{{{{{\mathcal{O}}}}}}}}$ and the Green’s function describing the dynamics of *ψ* then acquires the form

4 $${G}_{\psi \psi }(\omega,k)=\frac{1}{\omega -{v}_{F}(k-{k}_{F})-{g}_{s}{G}^{-1}(\omega,k)},$$

where *G*^−1^(*ω*, *k*), assuming the role of the self-energy, is the two-point function of the composite fermionic operator ${{{{{{{\mathcal{O}}}}}}}}$, and *G*_*ψψ*_ can be shown to be properly normalised as the Green’s function of an elementary fermion^38^. The most important piece of the puzzle, the qualitative form of the complex self-energy at energies near the Fermi level, thus comes from holography. It is in this context that we compute the inverse Green’s function *G*^−1^(*ω*, *k*) appearing in Eq. (4). This is done by solving the Dirac equation on the curved gravitational background spacetime^39^.

In general, as it has been done for the model in this paper, the gravitational equations needed to compute the holographic Green’s function can only be solved numerically. Nonetheless, the qualitative behaviour of the low-energy Green’s function of such a fermionic operator can be obtained analytically and it is completely determined by the infrared properties of the theory, captured in the duality by the inner geometry of the gravitational spacetime. The electron self-energy obtained in this way is the one we use for the nodal holes near the Fermi surface within the framework of an emergent particle-hole symmetry, and it possesses the desired (*ω*, *T*)-scaling properties, i.e., power-law exponents matching the experimental ARPES data. In particular, this implies that in the class of Einstein-Maxwell-Dilaton geometries characterised by the dynamical critical exponent *z* = *∞*, and in the presence of a Fermi surface, the inverse Green’s function at *T* = 0 (the low-energy behaviour can be easily generalised to small non-zero temperature^21,40^) has to assume a low-energy form near *k*_*F*_^7^ given by

5 $${G}^{-1}(\omega,k)=g\omega {(-{\omega }^{2})}^{{\nu }_{k}-1/2}+\ldots,$$

where *g* is constant in the limit *ω*, *k* − *k*_*F*_ → 0. It is important to note that the *k*-dependent power *ν*_*k*_ is solely dictated by the IR of the theory and thus is independent of any of the UV details. The lifetime of the fermionic excitations near the Fermi surface are then described by

6 $${\Sigma }^{{''} }(\omega,k)=-\frac{\lambda {v}_{F}}{2}{({\omega }^{2})}^{{\nu }_{k}}+\ldots .$$

Theoretically, the constant $\alpha \equiv {\nu }_{{k}_{F}}$ in the exponent in Eq. (2b), depends on the two (dimensionless) parameters in the Dirac equation, the mass *m* and the charge *q*, that encode certain defining properties of the conformal field theory^7^. We keep the mass fixed close to the limit of *m* = − 1/2 of the range allowed for by semi-holography (−1/2 < *m* < 1/2)^38^. Then, varying only *q* as a function of the hole doping *p*, captures the doping dependence of the exponent *α*(*p*) seen in the experimental data, using the numerically obtained linear relationship *α* ≈ 1.9*q*. Finally, the value of *λ* (see Eq. (2a)) leading to a match with experiment at each doping level can be obtained by adjusting the strength of the coupling *g*_*s*_ between the fermion *ψ* and the conformal field theory.

We can see that Eq. (5) has the same qualitative form as the semi-holographic response (4) for *ω*, *k* − *k*_*F*_ → 0. Semi-holography thus only changes the UV of the theory, i.e. the constant *g*, but it does not change the emergent behaviour ${\Sigma }^{{''} }\propto {({\omega }^{2})}^{{\nu }_{k}}$. In this sense, while we showed that in our ‘bottom-up’ approach we possess enough tuning knobs so as to get a semi-holographic spectral function that closely resembles the one observed in the ARPES experiments, we do not know whether the values used do indeed correspond to a consistent theory of gravity. Nonetheless, our main point that the self-energy contains a momentum-dependent power is a universal feature of *z* = *∞* holographic calculations, independent of the particular UV completion applied. In fact, while the theoretical model proposed here is rooted in holography, any quantum critical theory with infinite dynamical exponent and a large-*N* suppression of higher-order correlation functions will be able to give rise to an effective correlation function of the same form as in Eq. (4)^7^.

## Supplementary information

Supplementary Information

Peer Review File

## Data Availability

The experimental datasets recorded and/or analysed, as well as the theoretical data generated during the current study are available from the authors on request.

## Code availability

The code generated during the current study are available from the authors on request.
