# Supplementary material for: Momentum-dependent scaling exponents of nodal self-energies measured in strange metal cuprates and modelled using semi-holography
Source: Nat Commun. 2024 May 29;15:4581. doi: 10.1038/s41467-024-48594-6 (PMC11522462; doi:10.1038/s41467-024-48594-6)
Supplement: Supplementary file 1 — Supplementary Information [file 41467_2024_48594_MOESM1_ESM.pdf]

Supplementary information: Momentum-dependent scaling exponents of nodal self-energies measured in strange metal cuprates and modelled using semi-holography

S. Smit,[1,*] E. Mauri,[2] L. Bawden,[1] F. Heringa,[1] F. Gerritsen,[1] E. van Heumen,[1]
Y.K. Huang,[1] T. Kondo,[3] T. Takeuchi,[4] N. E. Hussey,[5,6] M. Allan,[7] T.K. Kim,[8]
C. Cacho,[8] A. Krikun,[9] K. Schalm,[10] H.T.C. Stoof,[2] and M.S. Golden[1,11,†]

[1]*Van der Waals - Zeeman Institute, Institute of Physics, University of Amsterdam, Sciencepark 904, 1098 XH Amsterdam, The Netherlands*
[2]*Institute for Theoretical Physics and Center for Extreme Matter and Emergent Phenomena, Utrecht University, The Netherlands*
[3]*Institute for Solid State Physics, University of Tokyo, Kashiwa, Chiba 277-8581, Japan*
[4]*Energy Materials Laboratory, Toyota Technological Institute 2-12-1 Hisakata Tempaku-ku, Nagoya 468-8511, Japan*
[5]*High Field Magnet Laboratory (HFML-EMFL) and Institute for Molecules and Materials, Radboud University, Toernooiveld 7, 6525 ED Nijmegen, The Netherlands*
[6]*H. H. Wills Physics Laboratory, University of Bristol, Tyndall Avenue, Bristol BS8 1TL, United Kingdom*
[7]*Leiden Institute of Physics, Leiden University, Leiden, The Netherlands.*
[8]*Diamond Light Source, Harwell Campus, Didcot OX11 0DE, United Kingdom*
[9]*NORDITA, KTH Royal Institute of Technology and Stockholm University, Hannes Alfvéns väg 12, 106 91 Stockholm, Sweden*
[10]*Institute-Lorentz for Theoretical Physics, Leiden University, P.O. Box 9506, Leiden, Netherlands.*
[11]*Dutch Institute for Emergent Phenomena (DIEP), Sciencepark 904, 1098 XH Amsterdam, The Netherlands*

SUPPLEMENTARY NOTE 1: ELECTRON-PHONON SELF-ENERGY

The electron-electron (e-e) interactions being modelled using semi-holography are not the only factors contributing to the total self-energy of the carriers in the cuprates. Firstly, there is a general, upward offset that is frequency and temperature independent (but sample dependent), usually attributed to impurity scattering. Secondly, and particularly visible in the ARPES data at low temperatures, electron-boson interactions also play a significant role. To properly disentangle these effects from the purely e-e interactions, a frequency and temperature dependent phonon-mode was added to the two-dimensional PLL fitting routine described in the main text as Supplementary Equation (1). The complete 2-dimensional fit function then becomes:

$$\Sigma^{''}(\omega, T) = \Sigma^{''}_{imp} + \Sigma^{''}_{e-ph} + \Sigma^{''}_{PLL}, \tag{1a}$$

$$\Sigma^{''}_{PLL}(\omega, T) = \lambda \frac{[(\hbar\omega)^2 + (\beta k_B T)^2]^{\alpha}}{(\hbar\omega_N)^{2\alpha-1}}, \tag{1b}$$

$$\Sigma^{''}_{e-ph}(\omega, T) = \int \alpha^2 F(\omega^{'}) K(\omega, \omega^{'}, T) d\omega'. \tag{1c}$$

Here $\alpha^2 F$ is the boson spectral density that is taken to be Lorentzian, and the temperature dependence is described by the kernel $K(\omega, \omega^{'}, T)$, containing the Bose-Einstein and Fermi-Dirac distributions [1]. The necessity of adding the electron-phonon (e-ph) contribution is clear from comparing the two sets of fits shown in Supplementary Fig. 1.

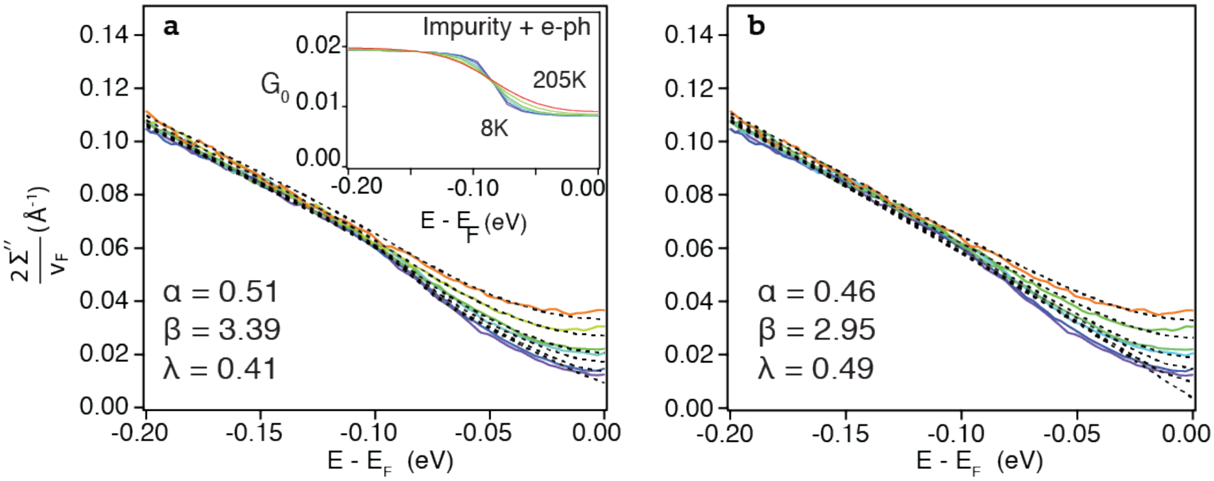

Supplementary Fig. 1. **Electron-phonon contribution to the self-energy.** Comparison of the 2-dimensional (ω, T) fits to the PLL phenomenology as described in the main text. **a:** With the e-ph contribution to the imaginary part of the self-energy and **b:** without the e-ph contribution. In the inset to panel **a**, the temperature dependence of the phonon self-energy is shown. The data are for a UD32K sample.

SUPPLEMENTARY NOTE 2: EFFECT OF A NON-LINEAR BARE BAND ON MDC LINESHAPES

Starting from the generic single-particle spectral function as given in Supplementary Eq. (2), there are two possible intrinsic reasons the MDC lineshape can deviate from being Lorentzian. Besides a momentum dependence of the self-energy, here in the power-law exponents as discussed in the main text, an asymmetry in the MDC lineshape could also be caused by an underlying non-linearity of the bare band: $\epsilon(k) \neq v_F(k - k_F)$ and

$$A(k,\omega) = \frac{1}{\pi}\frac{\mathrm{Im}\Sigma(k,\omega)}{(\omega - \varepsilon(k) - \mathrm{Re}\Sigma(k,\omega))^2 + (\mathrm{Im}\Sigma(k,\omega))^2} \ . \tag{2}$$

To examine whether this second option can be the source of the observed asymmetry or not, Supplementary Fig. 2 shows a simulated spectral function, generated with a momentum *independent* self-energy (given in panel **a**), with a reasonably-curved non-linear bare band (shown in red in panel **b**).

By 'reasonably curved' we mean that the bare band used is less than half-filled (or the Fermi level is closer to the band bottom at Γ than to the top of the band at the (π, π) points, so below the inflection point). For the hole-doped cuprates, this is always the case. In the example of Supplementary Fig. 2, a bare band is generated using the tight-binding model from Supplementary Eq. (3), with the following parameters: E_F = -0.44, t_0 = 0.7, t_1 = -0.11, t_2 = 0.03 (eV), and a = 5.3 $Å^{-1}$ [2], and

$$\varepsilon(\mathbf{k}) = E_F - \{2t_0\cos(k_x\frac{a}{2})\cos(k_y\frac{a}{2}) + 2t_1(\cos(k_xa) + \cos(k_ya)) + 2t_2\cos(k_xa)\cos(k_ya)\} \ . \tag{3}$$

Fitting the resulting MDCs to a symmetric Lorentzian, shows that the simulated spectral function (panel **d**) has more spectral weight at small momenta $|k < k_*|$, compared to the symmetric fit peaks, which is the opposite to what is observed in the experimental ARPES data shown in panel **c** of Supplementary Fig. 2 and that are presented and discussed in the main text. If a correction were to be considered for this, then the degree of asymmetry in the spectral function due to the k dependence of the self-energy would only increase.

For a greater-than-half filled band, the curvature could cause the MDC asymmetry to be similar to what is observed in the data. However, seeing as one always gets closer to the band bottom with increasing binding energy, a situation where the asymmetry is both in the correct direction *and* is increasing with energy is thus always incompatible with such a bare band.

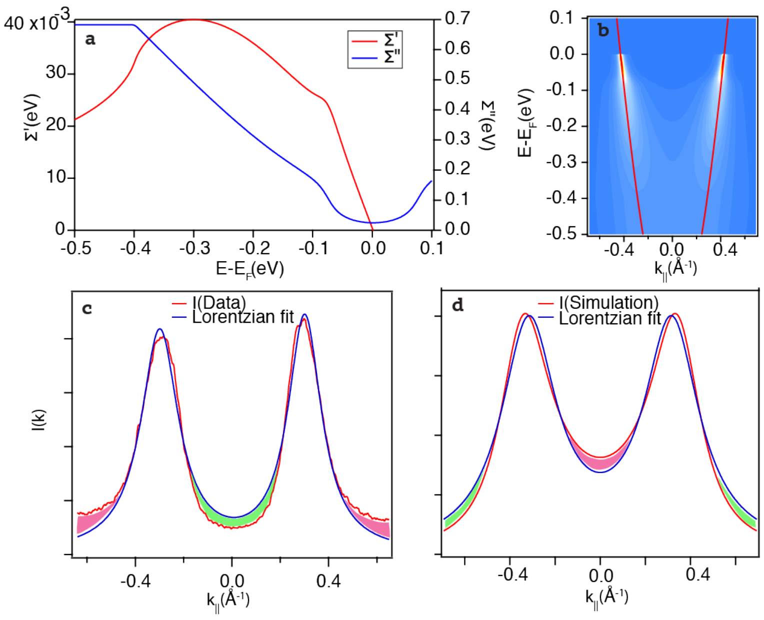

Supplementary Fig. 2. **The effect on the MDC lineshape from an underlying non-linear bare band.** **a**: Momentum-independent, Fermi-liquid-like self-energy used to generate a simulated spectral function shown in **b:** with a curved bare band indicated using a red line. **c:** An MDC through real ARPES data (from an OD15K sample at $E = 250$ meV) shown in red, including a fit with a pair of symmetric peaks (blue). **d:** An MDC through the simulated spectrum that includes the non-linear bare band, also overlaid with a fit using symmetric peaks. The difference between the fit and the data is highlighted in red where the fit underestimates the intensity, and in green where it overestimates it. Comparison of the fits to both the ARPES data in **c** and to the simulation in **d** shows that the observed asymmetry in the real data (higher intensity at $|k| > |k_*(\omega)|$), cannot be explained by an underlying non-linear bare band, and that the effects of the latter are, in fact, opposite to those we observe experimentally.

SUPPLEMENTARY NOTE 3: SURFACE INHOMOGENEITY/LOCAL DOPING VARIATION

The hole-doped cuprates, including $(Pb,Bi)_2Sr_{2-x}La_xCuO_{6+\delta}$, show a degree of real-space doping inhomogeneity [3], as well as non-perfect sample surfaces. In an ARPES experiment these effects are to an extent averaged over, as the source of photoelectrons (the lightspot) has a non-zero size of the order of tens of microns. Averaging over patches with surface normals pointing in slightly different directions will only lead to a net-broadening of the measured MDCs, as the measured MDCs are still symmetric around $k_{||} = 0$, whereby $k_{||}$ is the wavevector in the plane parallel to the sample surface.

To exclude local doping variation as the source of the observed asymmetry in the momentum distributions, we look at the renormalised dispersion in Supplementary Fig. 3 as determined by the position of the peak maxima k_* when fitting the MDCs to Lorentzians. Panel (b) shows that different (global) doping levels are easily distinguishable in our high-resolution data, as the position of the k_* moves to increasing $|\mathbf{k}|$ for underdoping at all energies.

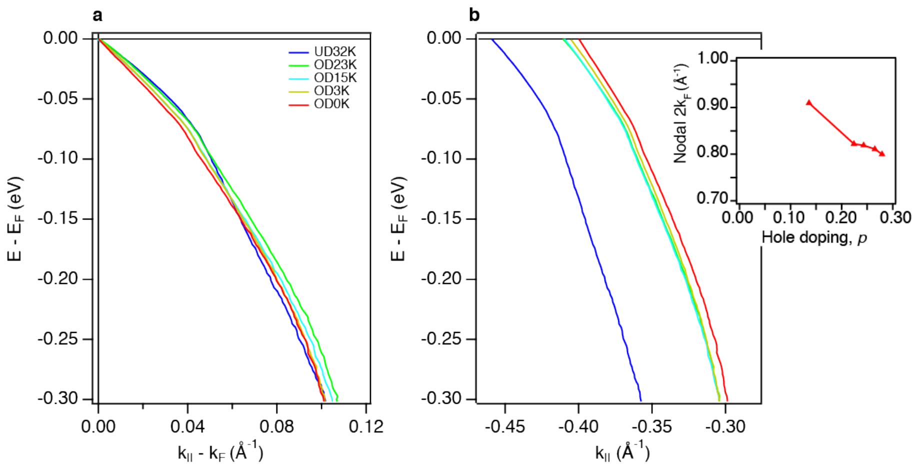

Supplementary Fig. 3. **Renormalised dispersions as function of doping.** (a) The MDC peak positions as function of energy and doping, relative to their respective k_F values. (b) The difference in absolute momentum between different global doping levels. In the inset the nodal k_F position is shown to decrease roughly linear with p

If each patch of differently doped sample were to contribute a symmetric MDC peak, it is imaginable that certain circumstances could cause asymmetric total lineshapes at fixed energy. Obviously any line shape can be fitted to an arbitrary number of Lorentzians with different positions and intensities, but a constraint here is that all the doping levels should have roughly similar widths for each energy (as can be seen in the main text in Fig. 1). A simulated distribution of patches, each patch contributing a Lorentzian MDC with similar widths but different ARPES intensities, that yield a combined lineshape similar to what is observed in the data is shown in Supplementary Fig. 4(a). However, from the doping evolution of the dispersion shown in Supplementary Fig. 3(b) and the inset thereof, it is immediately clear that such a doping distribution would also leave its mark at the Fermi level as an asymmetric, broad peak. This expected broad and asymmetric MDC at E_F is in stark contrast with the very sharp and symmetric peaks observed.

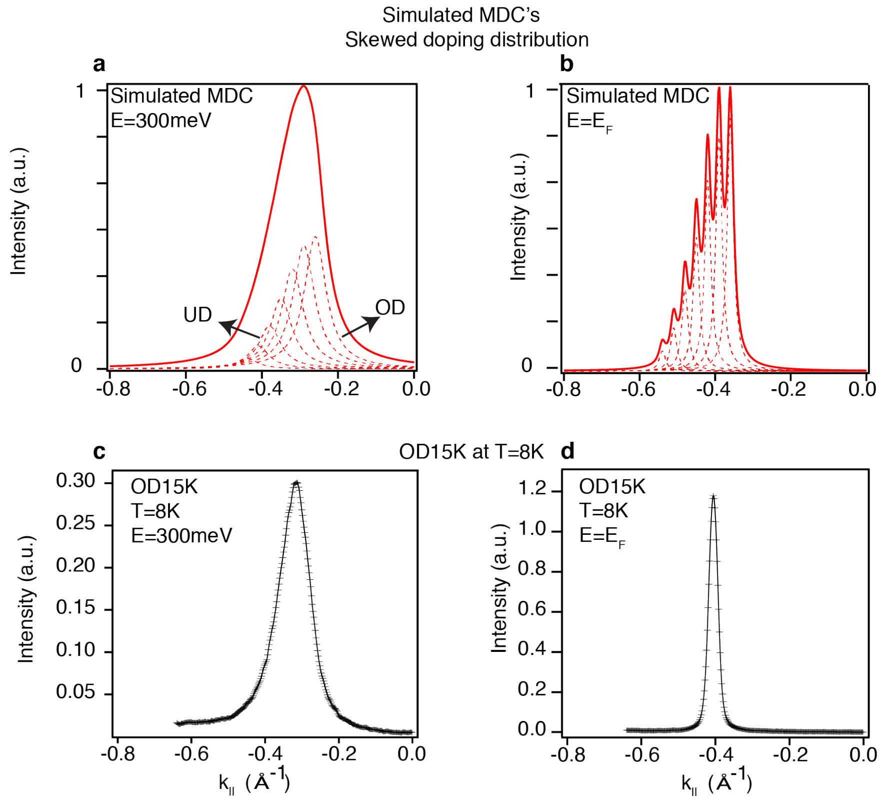

Supplementary Fig. 4. **Simulated lineshape stemming from local doping variation.**. (a) it is possible to generate an asymmetric lineshape (solid line) similar to the real data (c) from underlying symmetric Lorentzians of different intensities (dashed lines) stemming from differently doped, real space patches of sample probed in the ARPES experiment. However, such a distribution would also leave a clear mark in the Fermi level MDC shown in (b), as with increasing doping, k_F changes significantly. The fact that the observed MDCs at E_F are very narrow and symmetric (d) argues against such a scenario as the cause for the observed asymmetric lineshapes at non-zero binding energy.

SUPPLEMENTARY NOTE 4: DETECTOR INHOMOGENEITY

An inhomogeneous response of the multichannel plate (MCP), phosphor screen or - in principle - the air-side CCD in the detector system can lead to structured backgrounds in ARPES data, and these may distort lineshapes both in the energy (EDC) and angular (MDC) directions. By using the detector in ‘swept mode’, any energy-dependent background due to detector sensitivity is averaged out in the data-gathering stage.

To make sure no angle-dependent background distorts the final MDC lineshapes which are an important focus of this paper, I(E,k) data from an amorphous gold sample (Supplementary Fig. 5**a**) were measured with the same settings as the Bi-2201 samples (Supplementary Fig. 5**c**). The gold data show no dispersive features, and a close to constant density of states vs. energy close to E_F. As shown in (Supplementary Fig. 5**b**), the detector response is not completely flat in the angular (k) direction: the analyzer throughput efficiency or MCP sensitivity is a little higher in the center of the angular field of view than at the edges. We compared normalising the Bi-2201 data with the angular dependent profile in panel (b) or not, and these results - shown in (Supplementary Fig. 5**d**) - indicate that this has an almost negligible effect on the lineshape of the MDCs.

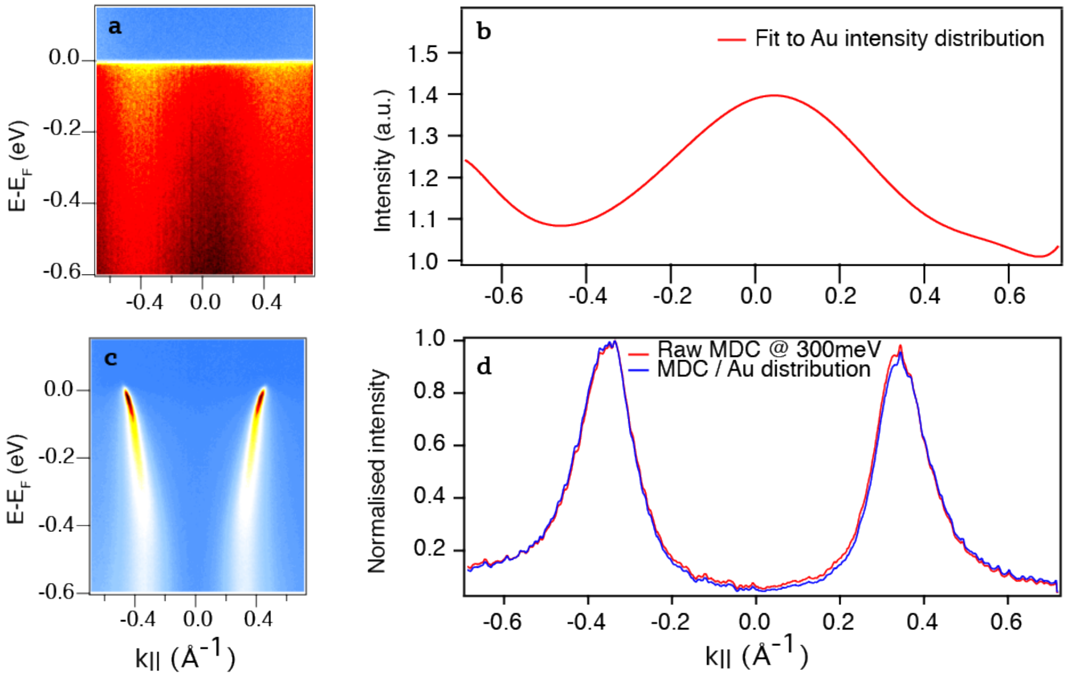

Supplementary Fig. 5. **a:** Measurement of an amorphous, clean Au film, with identical settings to those used to record the nodal Bi-2201 data in **c**. A slightly inhomogeneous detector response in the angular direction is visible, and in panel **b** a polynomial fit to the angle integrated Au I(E,k) image in panel **a** is shown. In **d**, Bi-2201 nodal MDCs at E=300 meV are also shown without and with division by the detector response function in panel **b**. It is clear that the MDC asymmetry is robust, remaining in both cases.

SUPPLEMENTARY NOTE 5: BACKGROUNDS

Before the fitting described in the main text is performed, a momentum-dependent background is subtracted from each EDM. This background is determined by integrating an energy window above the Fermi level, as illustrated in Supplementary Fig. 6

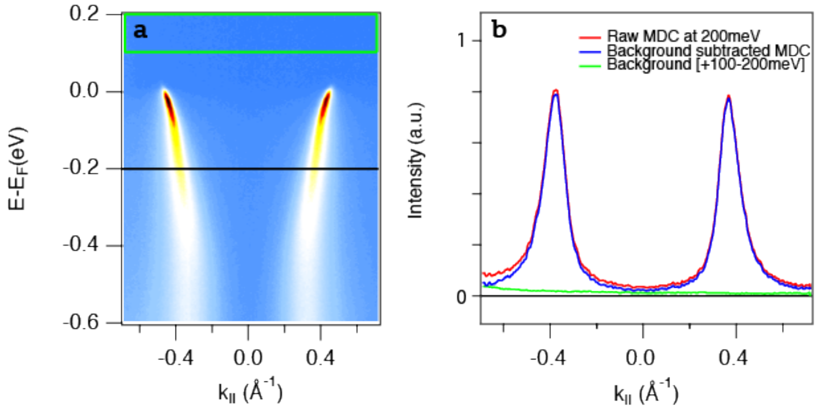

Supplementary Fig. 6. **Momentum-dependent background subtraction.** **a:** UD32K nodal ARPES I(E,k) image recorded at 8K. **b:** Example of the background subtraction carried out. An energy-independent background (green line) is acquired by averaging a window in energy between 100-200meV above the Fermi level indicated by the green box in panel **a**. The red curve in panel **b** is raw data, and the blue curve is obtained after subtraction of the background curve which itself is shown in green.

The fit function for each MDC also contains a constant offset A. This compensates for a possible incoherent, energy-dependent background, which is very small compared to the total signal, and does not influence the lineshape in the momentum direction. For both the Lorentzian and holography inspired fits, the total MDC fit function then becomes $I(\mathbf{k}) = \mathbf{A} + \text{Peak}_\mathbf{1} + \text{Peak}_\mathbf{2}$. A typical energy-dependent background generated this way is shown in Supplementary Fig. 7.

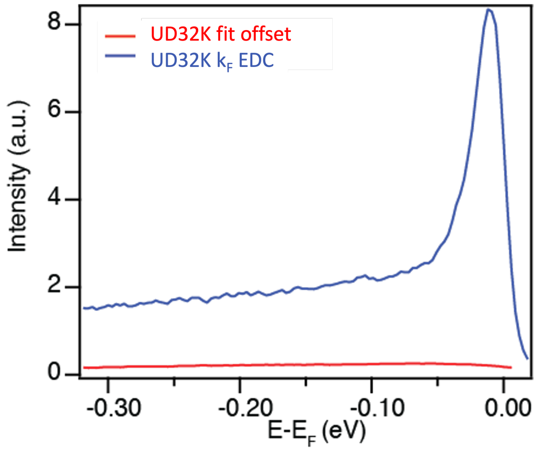

Supplementary Fig. 7. **Energy-dependent background.** The energy-dependent background compared to the k_F EDC of an UD32K sample measured at 8K.

SUPPLEMENTARY NOTE 6: ARTIFICIAL GENERATION OF ASYMMETRIC LINESHAPE BY ADDING AN ARBITRARY, STRONGLY-CURVED, POLYNOMIAL BACKGROUND

A key observation in this research is the fact that the peaks in the MDCs are increasingly asymmetric at growing energy below E_F. We have shown that this full energy-dependent behavior can be captured accurately by allowing for a k-dependent self-energy in the spectral function, of the form that is predicted by our holographic model, without increasing the number of free fit parameters beyond the PLL fits using symmetric Lorentzian fit functions. Supplementary Fig. 8 illustrates that the observed ARPES peak asymmetry can be manufactured when using symmetric Lorentzian peaks by the addition of an arbitrary, strongly curved background. Here, the green line shows a second-order polynomial added as a 'background' on which the two symmetric Lorentzian peaks are added. Not surprisingly, the residual is low, as the number of free fit parameters has expanded considerably. Apart from the principle point that we should not add further parameters to a fit unnecessarily (Occam's razor), we stress that the real data provide no justification at all for such a strongly curved background. Both the data from Bi-2201 show a background signal in Supplementary Fig. 6 unlike the green line in Supplementary Fig. 8, and the angular response function of the detector measured on a clean polycrystalline gold film in Supplementary Fig. 5a&b does not show any support for this type of angular variation of the intensity.

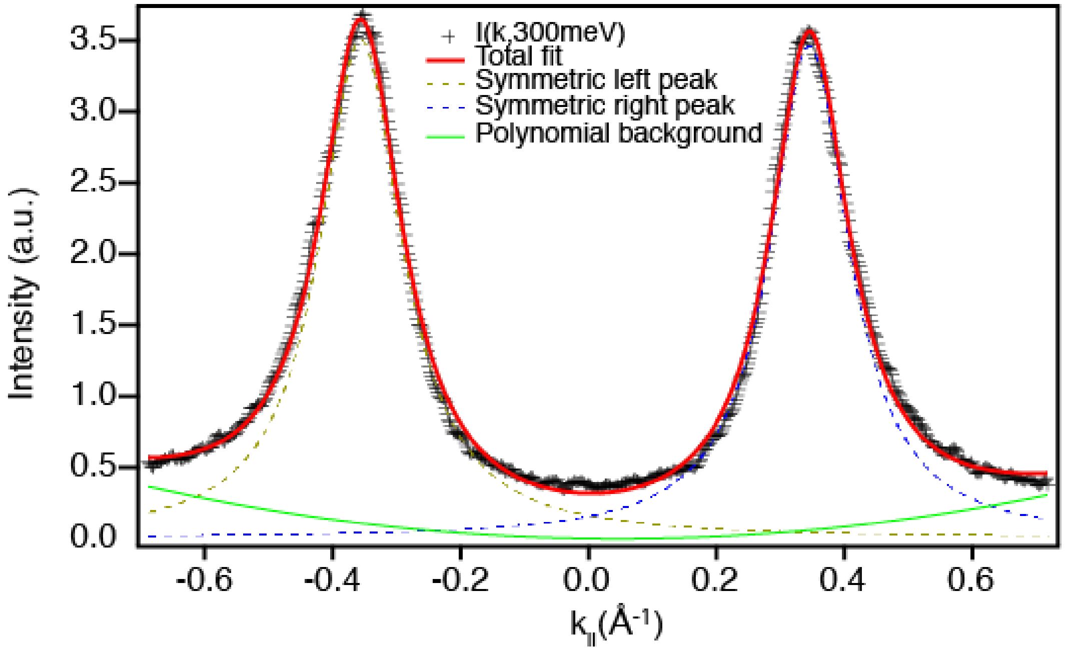

Supplementary Fig. 8. **Manufacturing the observed asymmetry of the MDC peaks by adding an arbitrary, strongly-curved background.** Shown is an ARPES MDC (crosses) recorded at an energy of 300meV from an UD32K sample measured at 8K, after background subtraction as described in Supplementary Fig. 6. The MDC can be fit (red) using a combination of two symmetric, Lorentzian peaks (dashed lines), if a strongly-curving polynomial background (green) is added. The ARPES data themselves present no independent evidence of such a background in the signal or detector response.

SUPPLEMENTARY NOTE 7: TEMPERATURE DEPENDENCE

Plotting and labelling *all* the measured temperatures for the different samples in Fig. 1 in the main text would be impractical. Here all the measured temperatures are listed in Supplementary Table I below. To prevent cluttering of Fig. 1 in the main text, not all the temperatures are shown in the figure, even though they are included in the fit and thus help determine the PLL fit parameters given.

**Sample**	Hole doping p	$T_{meas}(K)$
UD32K	0.14	**8**, **45**, **75**, **105**, **155**,180,**205**
OD23K	0.223	**8**, **75**, **105**,155,**180**, **255**, **295**
OD15K	0.243	**8**,30,**75**,105,**155**,180, **205**, **255**, **295**
OD3K	0.265	**8**,45,**90**,130,**155**, **180**, **205**, **230**
OD0K	~0.29	**8**,75,**105**, **155**,180,**205**, **255**, **295**

Supplementary Table I. Summary of the measured temperatures for each doping level, the temperatures that are shown in Fig. 1 are highlighted in bold. In total 39 temperatures, spread over 5 doping levels were recorded.

In the top two rows of Supplementary Fig. 9, an overview of the ARPES dispersion images I(E,k) for the lowest (upper row, **a**) and highest temperatures (middle row, **b**) for each sample is given. The lowest row, Supplementary Fig. 9**c** shows the asymmetry parameter V from fits using Equation (3) in the main text in which V was allowed to vary for each value of ω, as described in the main text discussing Fig. 4b. In this supplementary figure not only the low-T data but also those at the highest temperature for each sample are shown. The asymmetry parameter extracted in this manner does not show a clear temperature dependence. In panel **D** we show again the the β parameter extracted from the PLL fits (main text Fig 1), as well as the theoretical prediction from the Gubser-Rocha model.

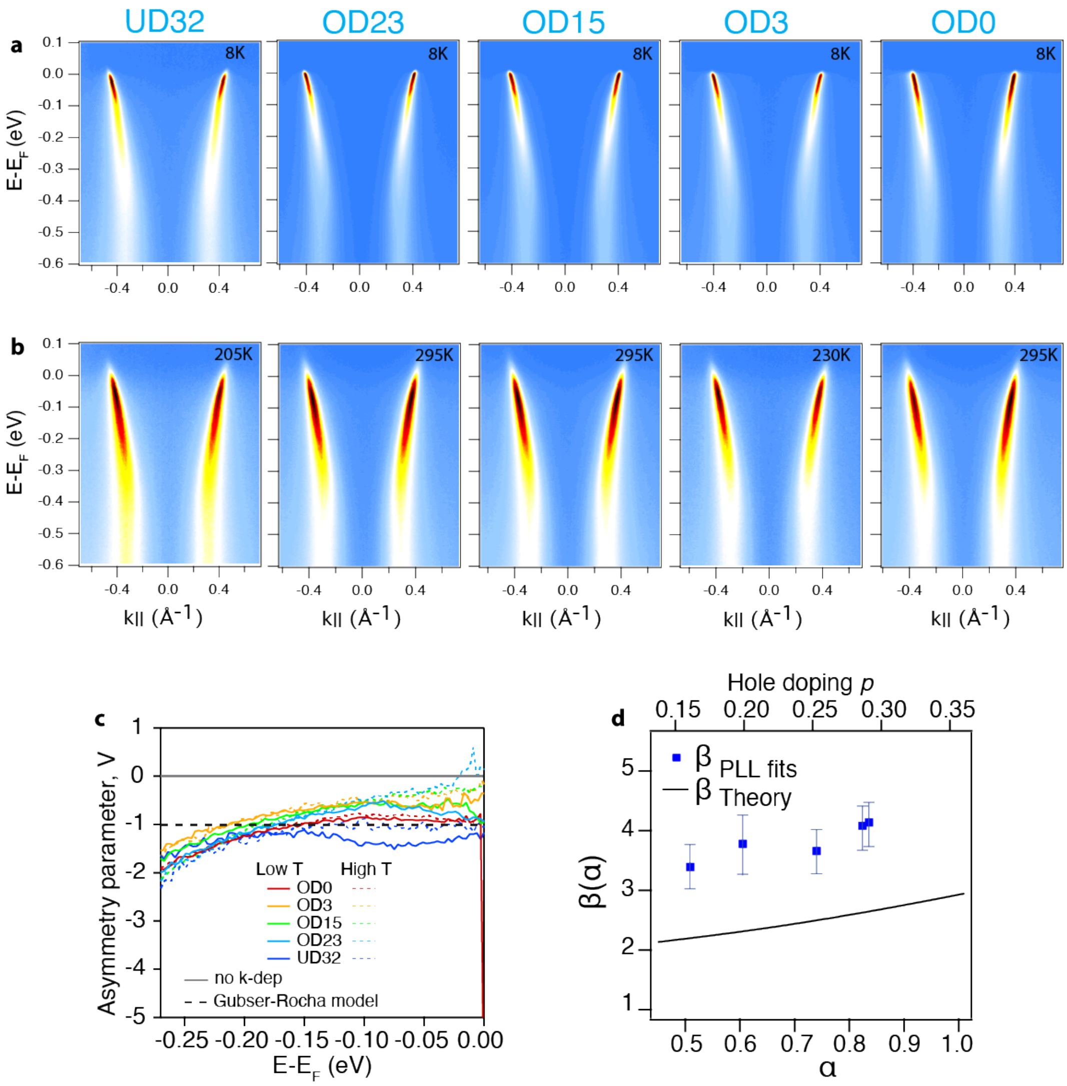

Supplementary Fig. 9. **Temperature dependence of the asymmetry. a:** Lowest and **b:** highest temperature nodal dispersions I(E,k) for all the measured dopings. The asymmetry parameter V is shown in **c**, using solid lines for low temperature, and dashed for high temperature data. These parameters come from fits to the data using Equation 3 in the main text. The β parameter, both those from the PLL fits in the main text and the prediction by the Gubser-Rocha model is shown in **d**. No clear temperature dependence is discernible.

SUPPLEMENTARY NOTE 8: MODEL-INDEPENDENT PARAMETRIZATION OF THE SELF-ENERGY: LINEAR EXPANSION IN k-k$_F$

In the main text the focus lies on describing the observation of asymmetric MDC peaks with a momentum dependence in the self-energy, using the specific prediction of our particular holographic model that it is the scaling exponent that is k dependent. A possible model-independent approach would be to linearly expand the self-energy around k_F, preserving the condition that $\mathrm{Re}\Sigma(k_F, E_F) = 0$,

$$\Sigma(k,\omega) = \Sigma(\omega) + (k - k_F)\partial_k\Sigma(\omega). \tag{4}$$

Substituting this for the self-energy, the spectral function is then given by:

$$A(k,\omega) = \frac{1}{\pi}\frac{\mathrm{Im}\Sigma(\omega) + (k-k_F)\partial_k\mathrm{Im}\Sigma(\omega)}{(\omega - v_F(k-k_F) - \mathrm{Re}\Sigma(\omega) - (k-k_F)\partial_k\mathrm{Re}\Sigma(\omega))^2 + (\mathrm{Im}\Sigma(\omega) + (k-k_F)\partial_k\mathrm{Im}\Sigma(\omega))^2}\ . \tag{5}$$

The k dependence at each energy ω can then be parametrized by a factor Γ^1 that is added to the Lorentzian fit function, as given in Equation (6):

$$L_{mod}(k) = \frac{W}{\pi}\frac{\frac{\Gamma^0 + (k-k_F)\,\Gamma^1}{2}}{[k-k_*]^2 + \left[\frac{\Gamma^0 + (k-k_F)\,\Gamma^1}{2}\right]^2}\ . \tag{6}$$

Including an additional free parameter to any fit function increases the quality of the fits, and the residuals are also low in this case. A non-zero value of Γ^1 indicates the presence of asymmetry in the fitted peak, and its magnitude signals the amount of k dependence at that energy. Neglecting the intensity component $W(\omega)$, comparison of Equations (5) and Equation (6) leads to the following relations between the physical and fitting parameters:

$$\mathrm{Im}\Sigma(\omega) \propto \frac{\Gamma^0}{2}, \qquad \partial_k\mathrm{Im}\Sigma(\omega) \propto \frac{\Gamma^1}{2}\ . \tag{7}$$

The Γ^0 and Γ^1 parameters for all dopings and temperatures are given in Supplementary Fig. 10. In general, the asymmetry in MDC peaks is very small for low energies (<100meV), tending to zero when approaching the Fermi level. For energies >100meV, the Γ^0 and Γ^1 data from all doping levels show a more-or-less linear trend with energy. However, if a data collapse were to be sought, such that the $\Gamma^0(\omega)$ and $\Gamma^1(\omega)$ curves in Fig. 10, should superimpose, showing a single behaviour vs. ω and T, a significantly non-trivial ω- and T-dependent scaling function describing the weight of Γ_1 in the linear expansion would be required. The microscopic justification of such a ω-, T- and p-dependent function would be a challenge for theory.

If such a linear decomposition would be sufficient to capture the physics at work (as naturally some information is lost by neglecting higher-order terms of the momentum dependence), the data of Supplementary Fig. 10 can be used as a training ground for the development of theoretical approaches able to deliver the observed p-, T-, ω-, and k-dependence of the Γ^1 and Γ^0 components of the nodal self energy. We stress that the power-law liquid seen close to k_F and the significant momentum dependence of the self-energy developing at non-zero frequencies and larger $k - k_F$ values cannot currently be explained by any conventional condensed-matter theories we are aware of.

In the main text of the paper, it is shown how the semi-holographic theory only requires a single, known number (-1) for the asymmetry parameter V, which together with the PLL-pinned values of λ, α, β and $\hbar\omega_N$ keeps a tight rein on the free parameter space in the fits. Fig. 4b in the main text shows that when V is allowed to vary from -1, it does not stray far.

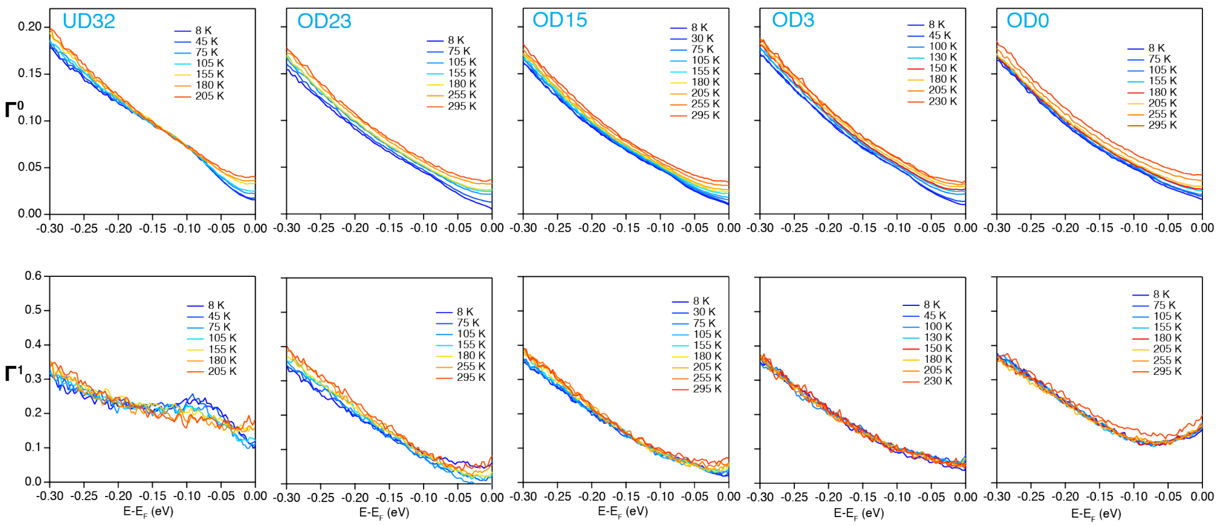

Supplementary Fig. 10. **Model-independent parametrization of the ARPES MDC asymmetry.** The k-independent part Γ^0 and the asymmetry parameter Γ^1, obtained by linear expansion of the Lorentzian width around k_F using Equation (6), plotted as function of energy for all dopings and temperatures measured.

SUPPLEMENTARY REFERENCES

* steef.smit@ubc.nl

† m.s.golden@uva.nl

[1] Cuk, T. *et al.* A review of electron-phonon coupling seen in the high-Tc. Superconductors by angle-resolved photoemission studies (ARPES). *Physica Status Solidi (B) Basic Research* **242**, 11–29 (2005).

[2] Berben, M. *et al.* Superconducting dome and pseudogap endpoint in Bi2201. *Physical Review Materials* **6**, 044804 (2022).

[3] Alldredge, J. W. *et al.* Evolution of the electronic excitation spectrum with strongly diminishing hole density in superconducting $Bi_2Sr_2CaCu_2O_{(8+\delta)}$. *Nature Physics* **4**, 319–326 (2008).
